# A vision transformer model for the detection of glaucoma from optic disc photographs

**DOI:** 10.1038/s41598-026-44662-7

**Published:** 2026-03-24

**Authors:** Ella Bouris, Brayden K. Leyva, Ojo Perpetua Odugbo, Jericho Lawson, Sang Wook Jin, Zhe Fei, Esteban Morales, Omar Alkhalili, Joseph Caprioli

**Affiliations:** 1 Glaucoma Division, Stein Eye Institute, Los Angeles, CA USA; 2 Department of Ophthalmology, Dong-A University College of Medicine, Busan, Republic of Korea; 3 Glaucoma Division, Stein Eye Institute, David Geffen School of Medicine, University of California at Los Angeles (UCLA), Los Angeles, CA 90095 USA; 4 Computer Science, University of California Los Angeles, Los Angeles, CA USA; 5 Department of Statistics, University of California, Riverside, USA; 6 Department of Ophthalmology, Faculty of Clinical Sciences, College of Health Sciences, University of Jos, Jos, Nigeria

**Keywords:** Glaucoma, Vision transformer, Deep learning, Fundus photography

## Abstract

This study uses a deep learning algorithm to analyze optic disc photographs (ODPs) and classify eyes as glaucomatous or healthy based on optic nerve appearance. ODPs from three databases were independently graded by two glaucoma specialists. Images were preprocessed using the open-source language R and RimNet, a deep learning model for optic disc segmentation, to prepare inputs for training. The model was developed with Python based on Google’s Vision Transformer (ViT). After assessing the ODPs, the model provided an output between 0 and 1 to predict glaucoma likelihood (≥ 0.5 signified glaucoma). Model performance was evaluated using the area under the receiver operating curve (AUC), where 1 indicates perfect classification. A total of 1,432 glaucomatous eyes with a mean MD of − 2.09 dB were analyzed using the model. The model achieved AUCs of 1.00, 0.98, and 1.00 in the training, validation, and test phases respectively. Overall accuracy in test images was 0.987, sensitivity was 0.994, and specificity was 0.969 with grader labels as the ground truth. A later assessment using 956 advanced glaucomatous eyes (MD < − 6 dB) with a mean MD of − 11.71 reached 99.9% accuracy. The model demonstrated high accuracy in detecting glaucomatous optic nerve damage from ODPs. The model’s strong potential for early disease detection suggests that deep learning can be a valuable, cost-effective tool in glaucoma screening, especially in resource-limited regions. Our study demonstrates the potential of deep learning in providing accessible, early-stage glaucoma detection, supporting global efforts to prevent vision loss.

**Supplementary Information:**

The online version contains supplementary material available at 10.1038/s41598-026-44662-7.

## Introduction

Glaucoma is a progressive optic neuropathy characterized by optic nerve head cupping and retinal nerve fiber layer thinning with associated patterns of visual field loss. Globally, it is the leading cause of irreversible blindness and the second most common cause of moderate to severe irreversible visual impairment^1^. Glaucoma is especially prevalent in low- and middle-income regions where there is a lack of health infrastructure to support its diagnosis and treatment^2^. It is estimated that 50% of current disease is undiagnosed worldwide since glaucoma remains visually asymptomatic until severe stages, and the burden of disease is expected to increase globally as populations continue to age^3^.

Blindness from glaucoma can be prevented in most cases with early diagnosis and timely intervention. However, population screening is not currently recommended due in part to low disease prevalence and a lack of evidence supporting its cost-effectiveness^4^. The ideal population screening tool is low-cost with minimal false positives (i.e. highly specific) to limit strain on overburdened health systems. Measurements of intraocular pressure, while inexpensive, have proven unreliable to predict which patients will suffer damage from glaucoma; damage can occur even at physiologic pressures, and only a minority of patients with elevated intraocular pressures develop visual field loss from glaucoma^5–7^. The utility of optic coherence tomography (OCT) is impeded by high cost and rapidly evolving technology. Visual fields, while important for the management of glaucoma, often do not display any defects until irreversible retinal ganglion cell death has occurred^8–10^. Fundus photographs may be the most appropriate modality for population screening, as they are inexpensively obtained and relatively effective in diagnosing early disease.

An essential component of glaucoma diagnosis is the evaluation of the optic disc by a trained specialist. However, assessment of the optic disc is subjective and hampered by low inter- and intra-observer agreement, even among expert clinicians^11–13^. The requirement of trained interpretation further reduces their utility in regions with limited healthcare^14,15^. Automation of the analysis of fundus photographs with artificial intelligence may help address these challenges. In this study, we propose a deep learning approach for the detection of early glaucomatous damage from optic disc photographs.

Several deep learning approaches have been developed for automated glaucoma detection from fundus photographs, including CNN-based models by Ahn et al. (AUCs 0.93–0.94)^16^ and ResNet50 architectures by Christopher et al. (AUC 0.91)^17^. These models however face significant limitations as most have been trained on mixed cohorts of early and advanced disease, with performance consistently lower in early versus advanced glaucoma. To address this gap, we developed a Vision Transformer (ViT)-based model specifically trained on early glaucoma (MD > − 6 dB), with the goal of achieving clinically acceptable accuracy and high specificity suitable for population screening programs in under-resourced regions.

## Methods

Optic disc photographs (ODPs) were selected from three databases: the clinical database at the Jules Stein Eye Institute at the University of California Los Angeles (UCLA), and the publicly available ACRIMA and RIM-ONE databases. The current study was a retrospective study, carried out in accordance with the tenets of the declaration of Helsinki and the Health Insurance Portability and Accountability Act (HIPAA) and was approved by UCLA’s Human Research Protection Program. All patient data was deidentified, and the cohort involved no more than minimal risk to the participants and did not adversely affect their rights and welfare. This study was not a clinical investigation subject to US Food and Drug Administration regulations. Considering these conditions, the UCLA institutional review board guidelines did not require an informed consent to be obtained from the participants of this study.

ODPs from all three datasets were classified independently by two glaucoma specialists; any instances of disagreement were adjudicated by a third expert grader. Eyes were classified as either glaucomatous or healthy based on the appearance of the optic nerve without reference to visual fields or the contralateral eye. Eyes with any concurrent retinal pathology that might confound the diagnosis of glaucoma were excluded.

### UCLA database

One thousand four hundred eighty-two ODPs from 1432 glaucomatous eyes and 50 healthy eyes were selected from the UCLA clinical database, collected from 1997 to 2021. ODPs from eyes with glaucoma were required to have a visual field with mean deviation (MD) value > -6 decibels (dB) within one year of the date of disc photograph capture. Eyes with MD < -6 dB were excluded to avoid eyes with moderate to severe damage from glaucoma and instead prioritize diagnostic accuracy in early disease. ODPs from healthy eyes were obtained from a dataset collected for a previous research study by the same investigators and belonged to individuals recruited as normal (non-glaucoma) control participants. These patients were required to have open angles, corrected visual acuity of 20/25 or better, normal visual fields, and no evidence of optic nerve damage on examination^18^.

All 1,432 included images were of usable quality based on a neural network previously developed at our institution^19^. The ODPs were a mix of digitized scanned slides. The slide images were captured with a Zeiss Fundus Flash 3 Camera on Kodachrome 25 film and commercially digitized. The digital images were captured on either a Zeiss Fundus Flash 3 with an Escalon Digital Back or a Zeiss FF450 with Digital Back.

We supplemented our 50 healthy control eyes with 605 images from two publicly available datasets, ACRIMA and RIM-ONE, described below. Only eyes from the UCLA Database were used for the glaucomatous group.

### ACRIMA database

The ACRIMA database is composed of 705 ODPs, 396 glaucoma and 309 healthy, based on expert exam and clinical findings. They were obtained through the ACRIMA project (TIN2013-46751-R) funded by the Ministerio de Economía y Competitividad of Spain with the approval of their local ethical board and in adherence to the Declaration of Helsinki. All images were captured with the Topcon TRC retinal camera and IMAGEnet Capture System. Images with poor quality or artifacts were not included in the ACRIMA database. Two UCLA glaucoma specialists reviewed the 309 healthy eyes in their database for any signs of glaucomatous damage or other retinal pathology. Twelve were excluded based on these criteria (six for a suspiciously thin neural rim, six for pathologic myopia), which left 297 healthy eyes for inclusion in our study.

### RIM-ONE database

The RIM-ONE database is part of a research project developed in collaboration with three Spanish hospitals: Hospital Universitario de Canarias, Hospital Clinico San Carlos, and Hospital Universitario Miguel Servet. It is composed of 485 ODPs, 313 of which are from healthy eyes. All are nonmydriatic images captured with a Nidek AFC-210 with a body of a Canon EOS 5D Mark II. Two UCLA glaucoma specialists reviewed the 313 healthy eyes in their database for any signs of glaucomatous damage or other retinal pathology. Five were excluded based on these criteria (all for suspiciously thin neural rims), which left 308 healthy eyes for inclusion in our study.

### Image pre-processing

All images were cropped around the disc with the disc being approximately 40% of the image area on average. This standardization was implemented to achieve relative uniformity across the three datasets, as the ACRIMA and RIM-ONE images are only available pre-cropped around the disc. The disc-to-image ratio varied naturally due to differences in optic disc sizes and disc localization. Disc areas were approximated using a circular area. The discs of the ACRIMA and RIM-ONE images were manually outlined with a circle and then cropping was applied as needed. The discs of the UCLA images were outlined using RimNet, a deep learning rim and cup segmentation model previously developed by our institution^20^. The center of the segmented rim and cup was used as the center of the disc. Next, we found the largest radius from the center to the edge of the segmented rim and cup. Using the center and calculated largest radius, a circle was drawn around the disc and then cropping was applied as needed. All images were checked for cropping errors.

Augmentations were applied using the Albumentations 1.3.0 Python library to the training images to increase sample size and reduce overfitting. Each augmentation was applied once to the glaucoma training group and twice to the normal training group to reduce the sample imbalance. Augmentations were limited to real world types of variations found in ODP. Augmentations were only applied to the training group after the images were randomly split into a training set, validation set, and testing set. Images were randomly rotated between 1 to 10 degrees or − 1 to − 10 degrees. Zoom was randomly applied from 0.05% to 20%. GaussianBlur was applied with a random Gaussian kernel size of 11 × 11 to 23 × 23. GaussNoise was applied with a random variance range for noise from 10 to 100. All images were resized to 224 × 224 with nearest neighbor interpolation.

### Model development

The model was developed with Python 3.10.11, employing a range of libraries including PyTorch 2.4.0, transformers 4.2.0, Pillow 10.4.0, OpenCV Python 4.6.0, Numpy 1.24.3, Scipy 1.10.1, and Albumentations 1.3.0. The model is based on Google’s Vision Transformer (ViT), which has been pre-trained on ImageNet-21 k at a resolution of 224 × 224^21,22^. The model first divides the input image into fixed-size patches, each of 16 × 16 pixels. Then it adds position embeddings to the patch embeddings, which retain spatial information about where each patch comes from in the original image. The core of the ViT model consists of a standard Transformer encoder architecture that includes 12 Multi-Head Self-Attention (MHSA) heads and a classification token to output the final classification result. The model has 86.4 million parameters.

This specific model can be imported from Python package transformers as the vit-base-patch16-224-in21k model. A batch size of 32 and an initial learning rate of 1e-5 were used. The full data were randomly split into train/validation/test sets with a ratio of 80/10/10. A total of 5 epochs were run on the training data, as the model converged fast and avoided overfitting. During training, we used a class-balanced sampler and class-weighted cross-entropy (i.e. focal loss) with weights proportional to the inverse class frequencies. fivefold cross-validation was performed by creating 5 separate folds of the training and validation data based on the original images, so that augmentations of the same image remain in the same fold and avoid data leakage.

For comparison, we fit a CNN-based transfer learning approach with ImageNet weights to initialize the EfficientNetV2B0 model^23^. A dropout layer and a fully connected dense layer were incorporated into the model with the following hyperparameter search: learning rate in 1e-2, 1e-3, and 1e-4; dropout rate in 0, 0.2, 0.4, and 0.6; and midlayer size in 0, 64, 512, and 1024. At this stage, the EfficientNet model was frozen to prevent further adjustments. We fine-tuned the model by unfreezing the three blocks of EfficientNet, namely block1, block4, and block6.

### Model output and outcome measures

The model’s output was a prediction score between 0 and 1 of the likelihood of the image being glaucomatous. An image with a prediction score of ≥ 0.5 was labeled glaucomatous.

The main outcome measure for the evaluation of model performance was the area under the receiver operating curve (AUC). The receiver operating curve (ROC) is a graphical representation of the trade-off between true positive rate and false positive rate at various decision thresholds. A perfect model will have an AUC of 1 and a poor classifier will have an AUC closer to 0.5. Overall accuracy, sensitivity, and specificity were also calculated for the model.

## Results

Table 1 provides a tabulation of the demographic and clinical characteristics of the 1432 glaucomatous eyes collected at UCLA. All eyes were primary open angle glaucoma (POAG). The mean MD was − 2.09 (± 1.91) dB. It was a racially heterogeneous dataset, composed of 55.8% Caucasian eyes, 14.0% Asian, 8.9% Black, 6.8% Hispanic, and 14.5% of unknown or other race. No additional data was available for the ACRIMA or RIM-ONE databases upon request to the respective database managers, so a comparison of the demographic and clinical characteristics of the two groups was not possible. Six images from ACRIMA were excluded due to the disc to image ratio exceeding 60%.

**Table 1:** Demographic and ocular characteristics for the UCLA glaucoma dataset.

Variable	n (%)
Sex	n (%)
Male	635 (44.3%)
Female	797 (55.7%)
Age (± SD*)	66.8 (± 11.4)
Eye	
Right	527 (36.8%)
Left	905 (63.2%)
Race	
Caucasian	799 (55.8%)
Asian	200 (14.0%)
Black	127 (8.9%)
Hispanic	98 (6.8%)
Other/Unknown	208 (14.5%)
Glaucoma diagnosis	
Primary open angle glaucoma	1432 (100%)
Mean deviation (± SD)	+ 2.04 (± 1.91)

*SD, standard deviation.

**Fig. 1:**
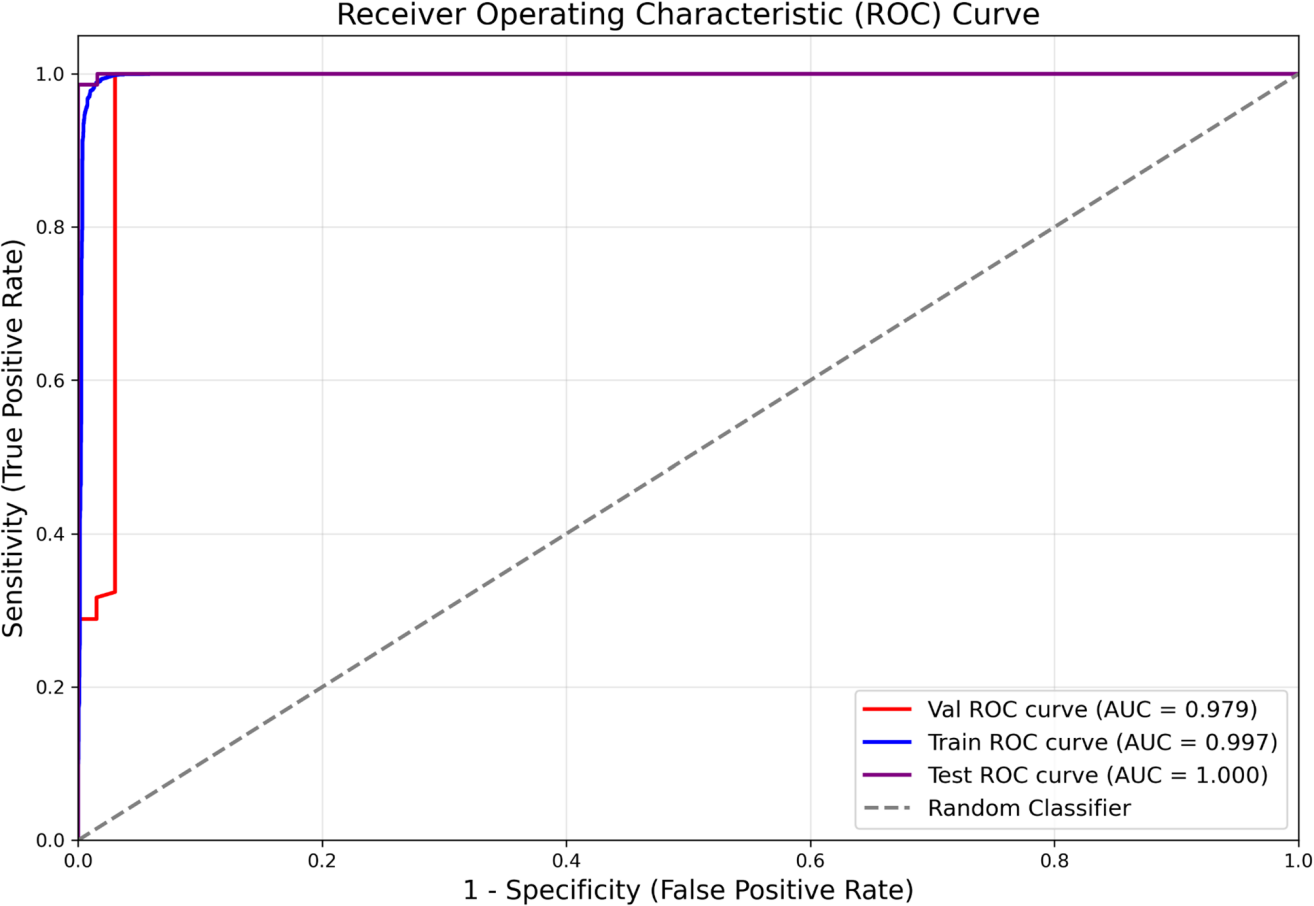
Receiver operating curves (ROC) on Train/Val/Test with ViT.

**Fig. 2:**
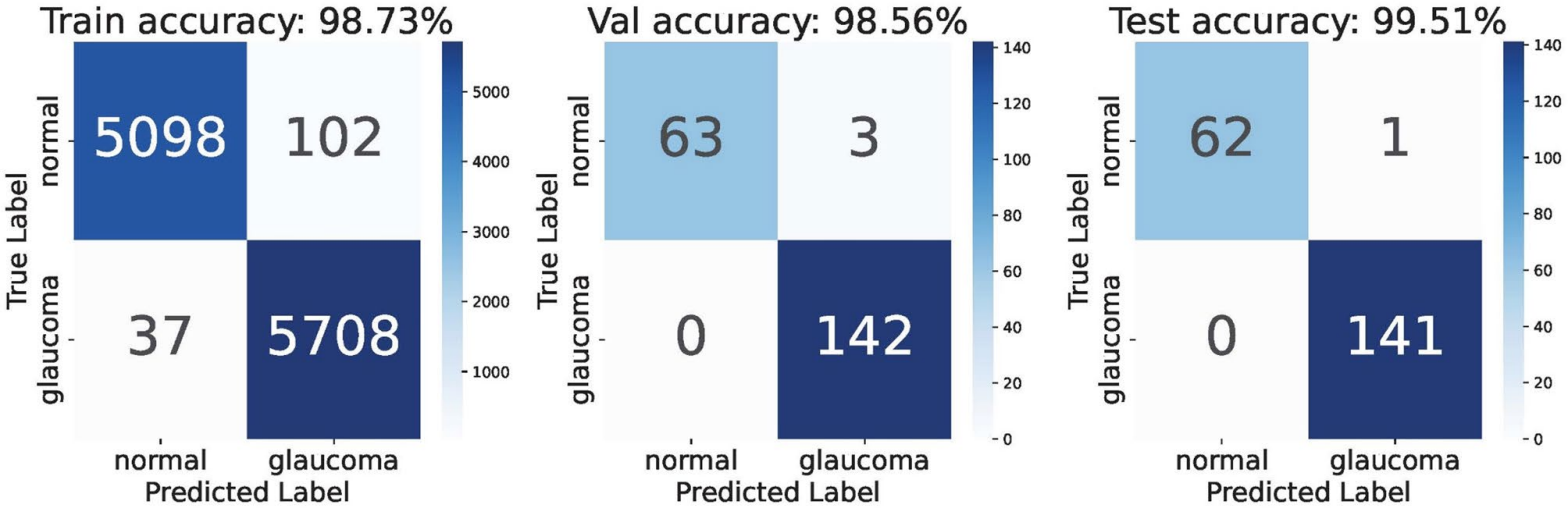
Confusion matrices depicting the model’s performance on the training, validation and test images. Ground truth is based on grader labels.

**Fig. 3:**
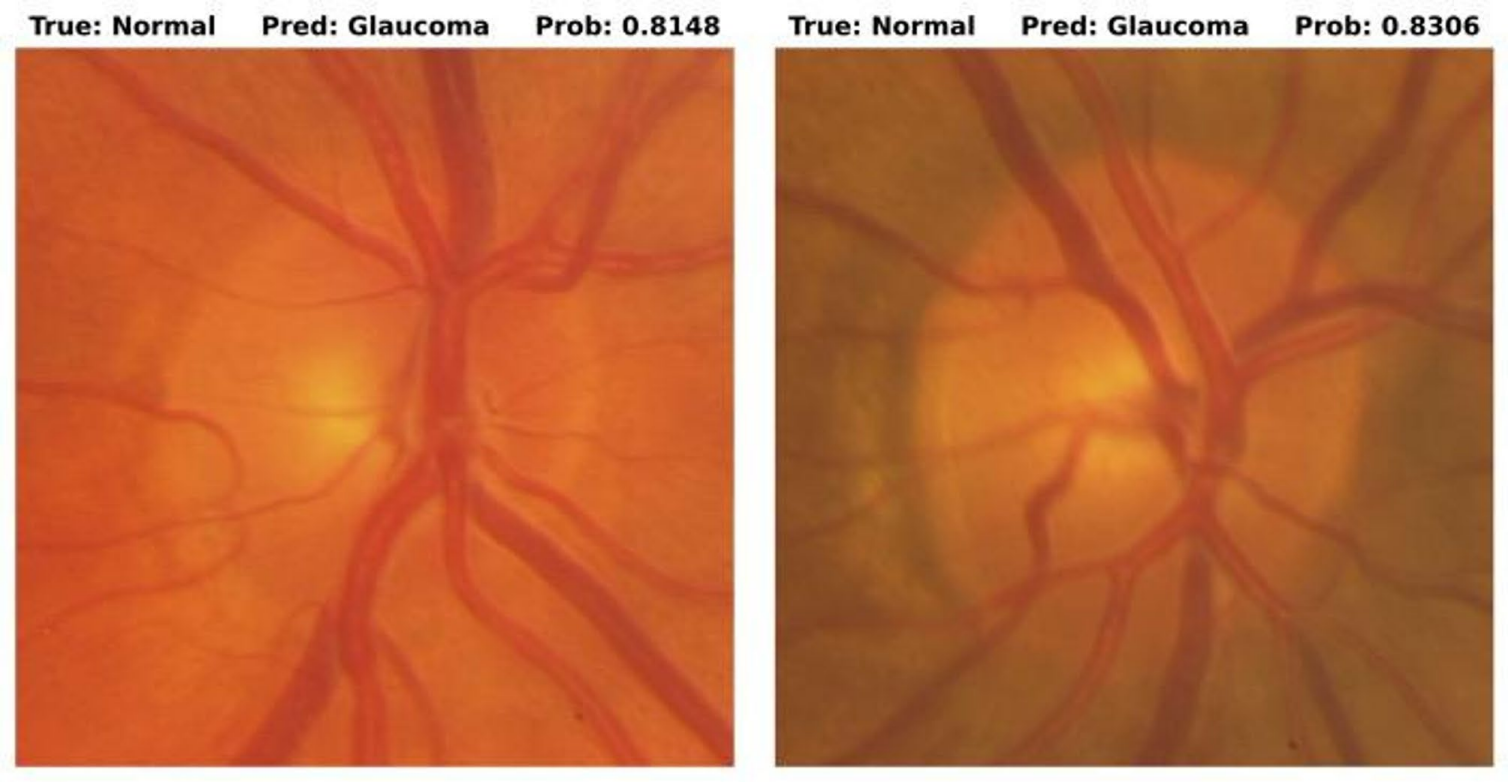
Examples of images mispredicted by the model along with the prediction score for each image. Left from the validation set, and Right from the test set.

With augmentations to increase the training size as described in Methods, we obtain training, validation, and test sets of 10,945 (5745 glaucoma, 5200 normal), 208 (142 glaucoma, 66 normal), and 204 (141 glaucoma, 63 normal) images respectively. The model achieved AUCs of 0.997, 0.979, and 1.00 across the three sets (Fig. 1). Overall accuracy in test images was 0.995, sensitivity was 1.0, and specificity was 0.984 with grader labels as the ground truth. The confusion matrix in Fig. 2 displays the model’s prediction summary on the training, validation, and test sets. There was 1 false positive (i.e. the model predicted glaucoma in a healthy eye) and no false negatives (i.e. glaucoma was not detected by the model) in the test images. Examples of mispredictions are shown in Fig. 3. The fivefold Cross Validation training yielded training, validation and test accuracies (Mean (SD)) as 98.73% (0.001), 98.56% (0.0034), 99.41% (0.004). The respective AUCs are 0.996 (0.002) for training, 0.982 (0.0024) for validation and 0.998 (0.002) for testing.

In comparison, the CNN-based transfer learning model with EfficientNet achieved a test AUC of 0.95, accuracy was 0.93, sensitivity was 0.96, and specificity was 0.85. Specifically on the test set, there were 10 false positives and five false negatives.

The model was subsequently tested on 956 eyes with moderate to advanced glaucoma (MD < − 6 dB) to ensure full practical utility. It achieved 99.9% accuracy on this dataset, which had an average MD of − 11.71, and mispredicted only one glaucomatous eye as healthy.

## Discussion

Our neural network demonstrated clinically acceptable accuracy at detecting early glaucoma based on a single optic disc photograph, with AUCs of 1.00, 0.98, and 1.00 across train/val/test sets (Fig. 1). Overall accuracy in test images was 0.987, sensitivity was 0.994, and specificity was 0.969 with grader labels as the ground truth. This suggests that deep learning methodologies may be a useful tool in the diagnosis of early glaucoma to facilitate timely intervention and blindness prevention and may be a viable option for population screening programs, particularly in under-resourced regions.

Several previous studies have developed a similar model for glaucoma diagnosis based on fundus photographs, however, only two reported information on the stage of disease in their patient cohorts, and none focused specifically on early glaucoma. Ahn et al. separated their cohort into 786 healthy eyes, 467 with advanced glaucoma (defined as near total cupping of the optic nerve, with or without severe visual field loss within 10° of fixation) and 289 with early glaucoma (glaucomatous retinal nerve fiber layer (RNFL) defects in red-free RNFL photography, without visual field defects)^16^. They tested a simple logistic classification model, a pre-trained model with GoogleNet Inception v3 backbone, and a convolutional neural network (CNN) of their own design and compared the model performances in classifying glaucomatous vs. healthy eyes. The logistic classifier is the only model for which they report the model’s ability to distinguish between early glaucoma and healthy eyes; an accuracy of 73% is cited as evidence that a more complex deep learning approach is required for the detection of early disease. However, results for the two deep learning approaches are reported only in the aggregate glaucoma (advanced and early stage) vs. healthy binary outcome, with no further subdivision by disease stage. The transfer learning model and their own CNN had accuracies of 84.5% and 87.9%, respectively, and AUCs of 0.93 and 0.94.

Christopher et al. evaluated the performance of several deep learning architectures with and without the use of transfer learning^17^. Their best-performing model, which used ResNet50 architecture with transfer learning, achieved an overall AUC of 0.91 in distinguishing glaucomatous optic neuropathy (GON) eyes from healthy eyes. The model’s AUC for identifying GON eyes with mild functional loss (MD > − 6 dB) was 0.89, compared to 0.97 for classifying eyes with moderate-to-severe functional loss (MD < − 6 dB). It had a sensitivity and specificity of 0.82 and 0.82 in identifying mild GON; both were higher for moderate-to-severe GON. Our moderately improved AUC and accuracy with respect to the previous literature may be attributed to the subset of early glaucoma fundus photographs used in training and validation.

Other studies have confirmed the relative difficulty of distinguishing between eyes with early or suspected glaucoma and healthy eyes as compared to more advanced disease. In a study by Phan et al., the models tested performed better (difference in AUC > 0.1) on images of eyes with confirmed glaucoma than on images with suspected glaucoma^24^. Zulfira et al. reported similar difficulties; their dynamic ensemble classifier performed well on cases of advanced glaucoma but frequently misclassified eyes with early glaucoma as healthy. The authors do not provide their criteria for early versus advanced glaucoma and state only that the model used cup-to-disc ratio and extent of peripapillary atrophy to make its classifications^25^. The findings in the aforementioned studies are not surprising. The difficulty discriminating between normal eyes and eyes with early glaucoma is well documented and can be accounted for in part by the variation in optic disc appearance in both normal and glaucomatous eyes^26–29^. We attempted to improve the detection of early glaucoma by training a neural network exclusively on disc photographs with an associated MD > − 6 dB and normal eyes. We achieved a similar or higher accuracy than other reports in the literature on eyes with both early and moderate-advanced glaucoma, demonstrating the utility of the model in this work.

Any screening tool must be able to detect disease with very high specificity to minimize the burdens associated with unnecessary referrals. False positive results often lead to further diagnostic tests and even unneeded medical or surgical interventions, which can be costly both to the individual and the healthcare system as a whole. Further, screening tools should be capable of highly accurate detection of early disease in particular, when interventions are likely to be more effective and the risk of visual impairment can be minimized.

The generalizability of any model is also particularly salient when considering the geographic areas to which this screening method may be applied, as many of these regions would include predominantly non-Caucasian eyes. Our neural network is likely to be more generalizable than other comparable models since it was trained on a racially heterogeneous dataset composed of individuals of white (59.4%), Asian (13.0%), African (8.5%), and Hispanic (6.8%) descent. For example, the dataset used by Christopher et al., while larger in size, was comprised of 95% European and African eyes and included very few other ethnic groups. This is particularly relevant when basing diagnosis solely on examination of the optic disc, as the disc morphology is highly variable between racial groups. Asian populations have a higher prevalence of myopia and are associated with a tilted optic disc phenotype, both of which can potentially confound accurate diagnosis of glaucoma based on optic disc appearance^30–32^. Conversely, mean optic disc area and cup area are larger and the optic disc cup is deeper in individuals of African descent than Caucasians; these morphological variations can mask rim thinning and similarly hamper accurate diagnosis of glaucoma in these patient populations^33–36^. In fact, pathologic myopia and physiologic cupping were two of the most common reasons for false-positive results in a similar study^37^.

This study is not without its limitations. Comparison of our own patient cohort with those in the publicly available databases was not possible because of the lack of demographic and clinical data available from the external datasets, therefore the possibility of significant differences, e.g., unknown racial heterogeneity in the ACRIMA and RIM-ONE datasets, between the populations cannot be ruled out. Additionally, although the variety of cameras among the datasets likely improved the generalizability of the model, the ODPs from external datasets (and therefore comprising most of the healthy cohort) were captured by different cameras than our own internal glaucomatous images and as such it is possible that this introduced bias into the algorithm. Due to the low number of healthy ODPs in our dataset and available public datasets, the number of glaucomatous ODPs included was limited to ensure an appropriately balanced dataset; as a result, our overall sample size was smaller than other comparable studies^17,37^ Deep learning networks have high data requirements, meaning that increasing the sample size could substantially improve model accuracy and generalizability^38^. Increasing the availability of high quality, disease-labeled datasets available for general use is crucial to addressing this limitation. Additionally, SMOTE or GAN-based augmentation techniques can be considered to address class imbalance.

Future work should aim to more closely simulate population screening conditions, including training or testing this model on images acquired with more limited imaging technology, e.g., a nonmydriatic, portable fundus camera, that is more representative of what may be feasibly used in a lower-resource screening setting. Additionally, previous studies have shown that poor-quality images or coexisting high myopia were common causes of false-positive and false-negative results^24,37^. While we excluded images in these categories from our model, both are likely to be present in real clinical scenarios. As such, their effects should be further investigated and quantified, as the ideal screening program should minimize unnecessary referrals to an already overburdened health system. Further, including basic patient data such as age, race, and refractive error may affect the model’s accuracy and should be explored. Integration of this model with similar algorithms capable of detecting ocular conditions such as diabetic retinopathy can also be explored as a way to further increase the utility and cost-effectiveness of disease screening programs^39–41^.

In summary, the present study demonstrates that deep learning can be applied to create an algorithm capable of detecting early-stage glaucoma with clinically acceptable accuracy in a racially heterogeneous dataset. It did so with an acceptably low false positive rate, which is important for a screening test, to limit the additional burden on existing health systems. With further validation on larger real-world datasets, this suggests that deep learning represents a possible pathway to implement cost-effective population screening programs to increase disease detection and reduce blindness from glaucoma, particularly in under-resourced regions.

## Supplementary Information

Below is the link to the electronic supplementary material.

Supplementary Material 1

## Data Availability

The data underlying this article are de-identified patient records and are available upon request to Dr. Joseph Caprioli, Ophthalmology, Jules Stein Eye Institute, Los Angeles, CA 90095, USA; Caprioli@jsei.ucla.edu.
