# Supplementary material for: A vision transformer model for the detection of glaucoma from optic disc photographs
Source: Sci Rep. 2026 Mar 24;16:14831. doi: 10.1038/s41598-026-44662-7 (PMC13168687; doi:10.1038/s41598-026-44662-7)
Supplement: Supplementary file 1 — Supplementary Material 1 [file 41598_2026_44662_MOESM1_ESM.docx]

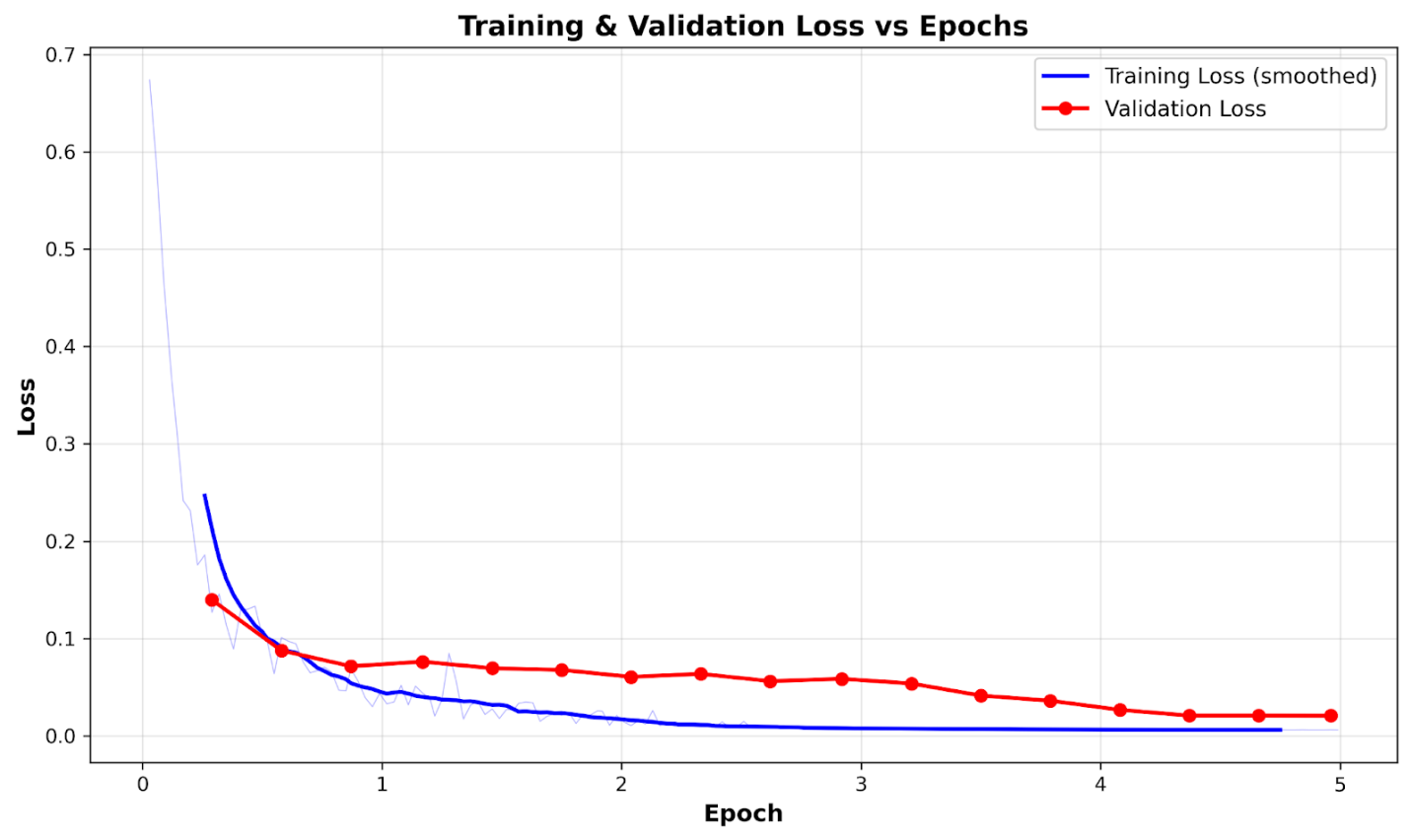


Supplement Figure 1. Training and validation losses versus epoch for our model.


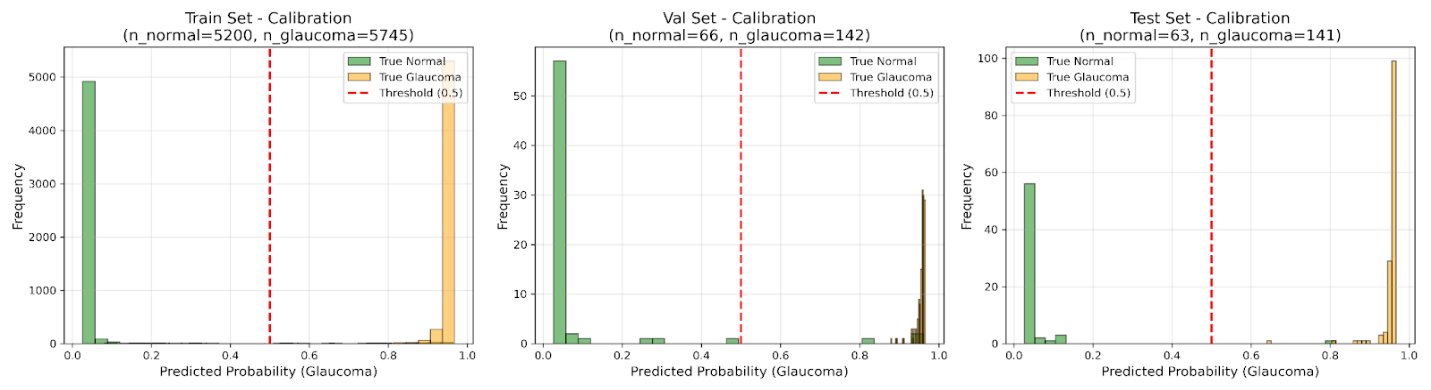


Supplement Figure 2. Histograms of predicted probabilities by true label.


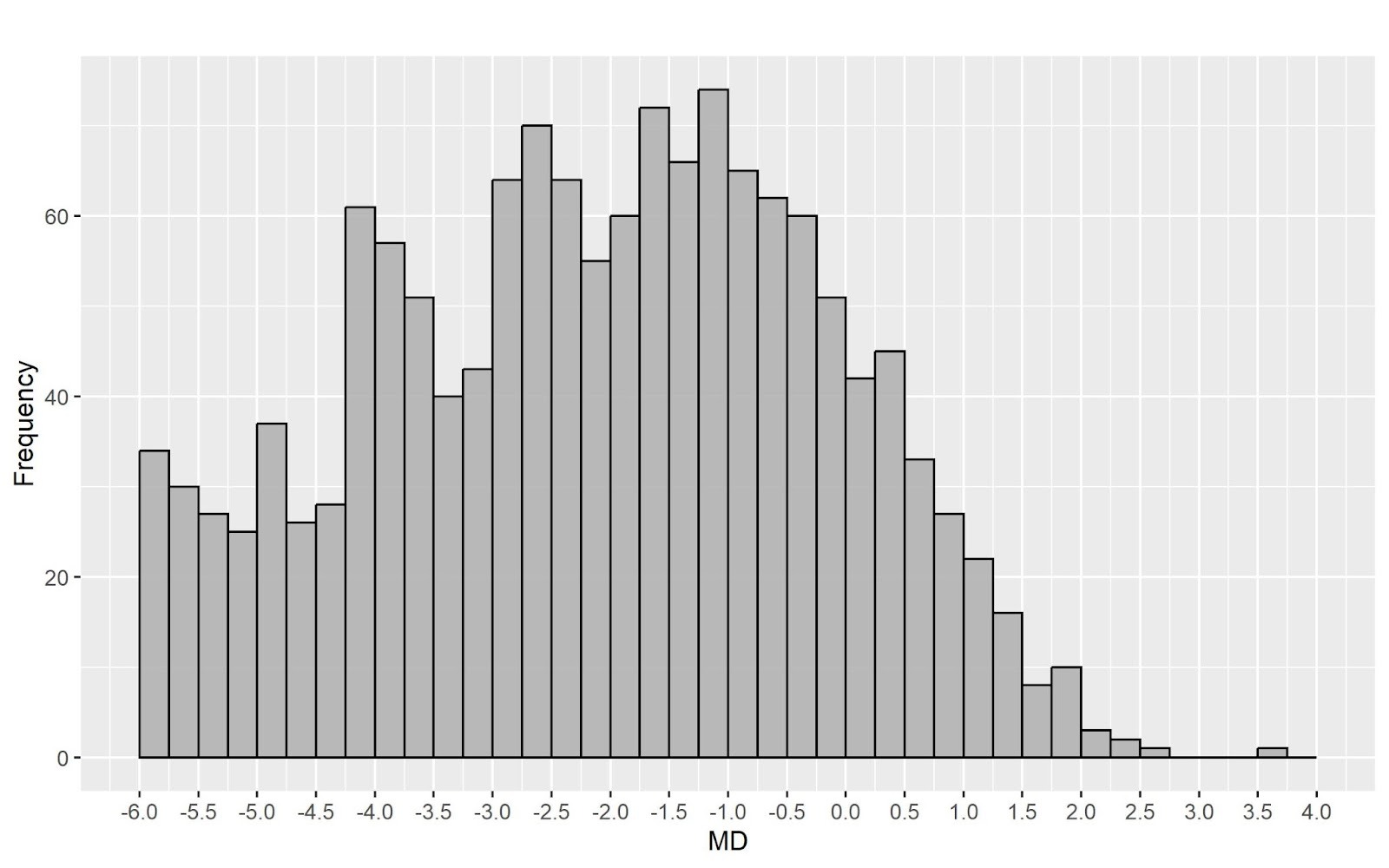


Supplement Figure 3. Distribution of the mean deviation within 1 year of the fundus photo for the UCLA glaucoma group.


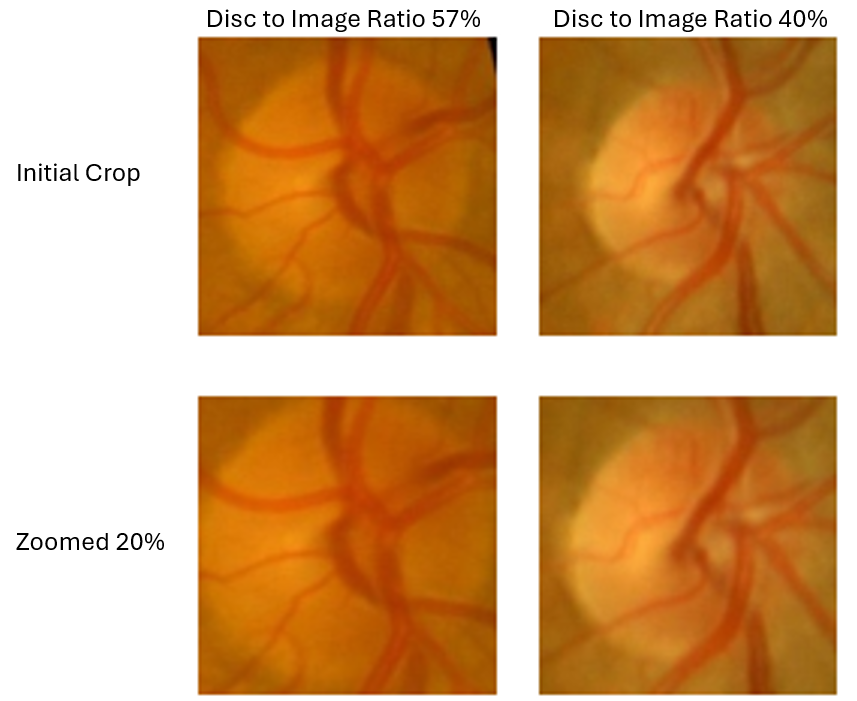


Supplement Figure 4. Result of applying 20% zoom, after the initial cropping, to the optic disc photographs with the highest and lowest disc to image area ratios in the dataset.


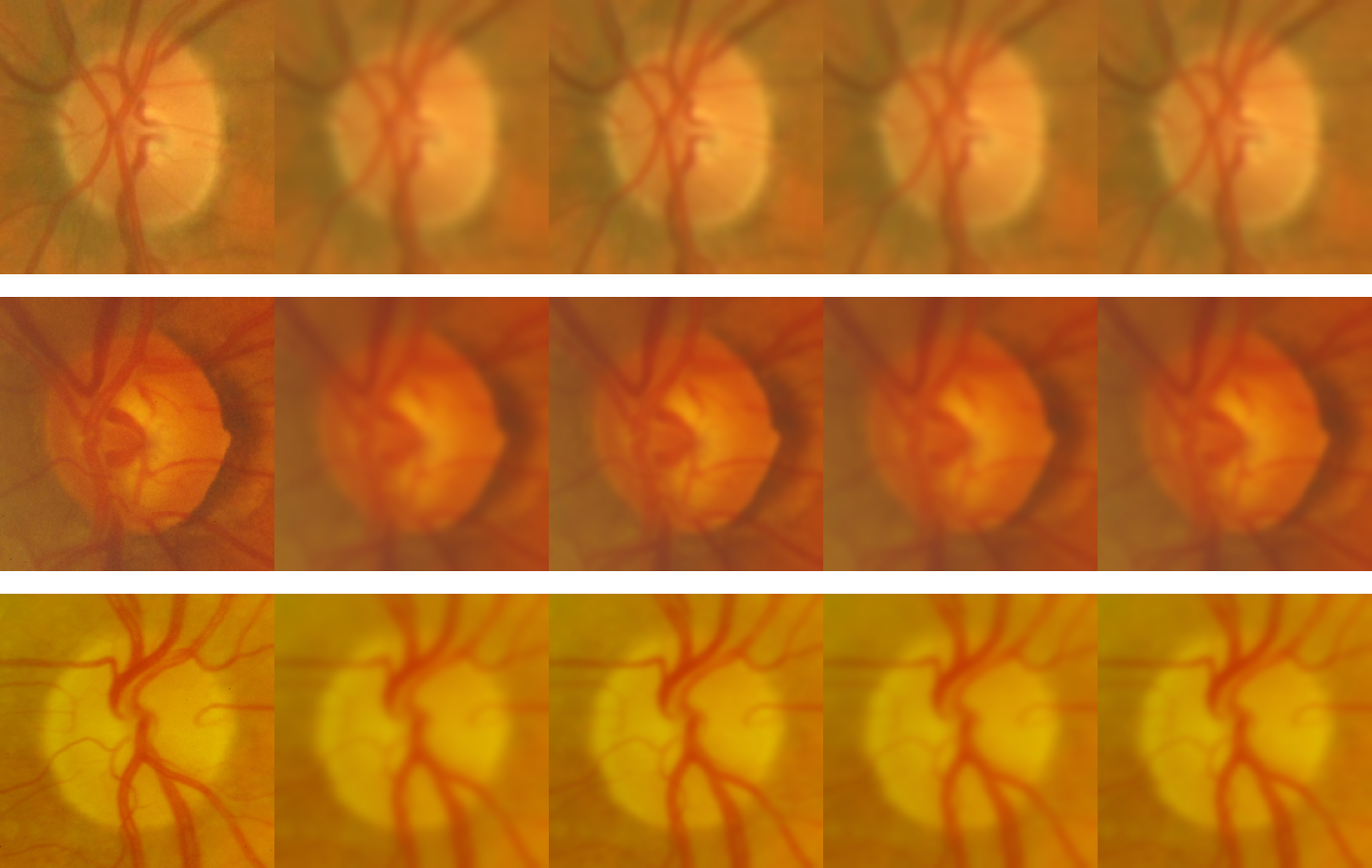


Supplemental Figure 5. Sample output of blur augmentation. The first column is the unaltered image. Subsequent columns are random applications of the augmentation.


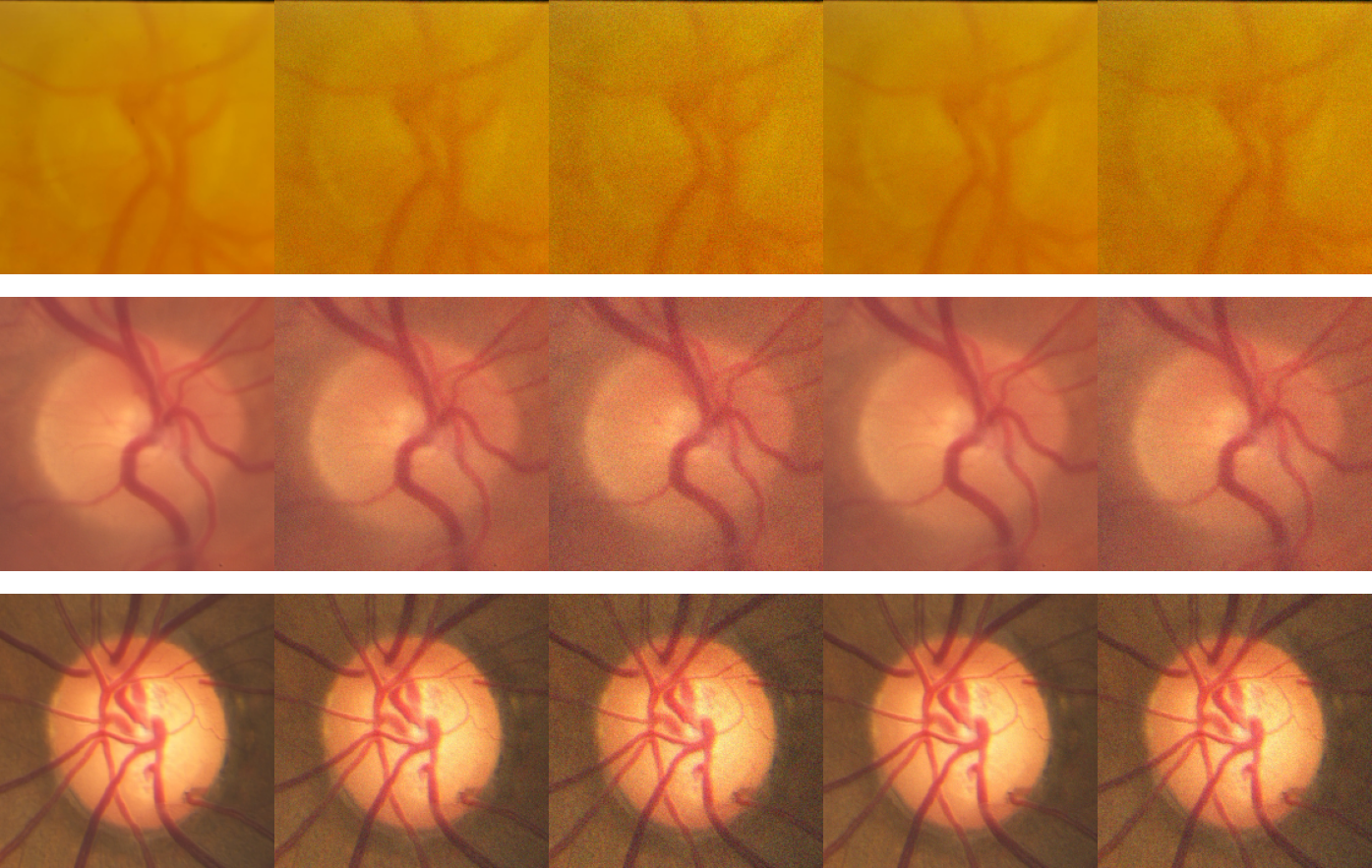


Supplemental Figure 6. Sample output of noise augmentation. The first column is the unaltered image. Subsequent columns are random applications of the augmentation.


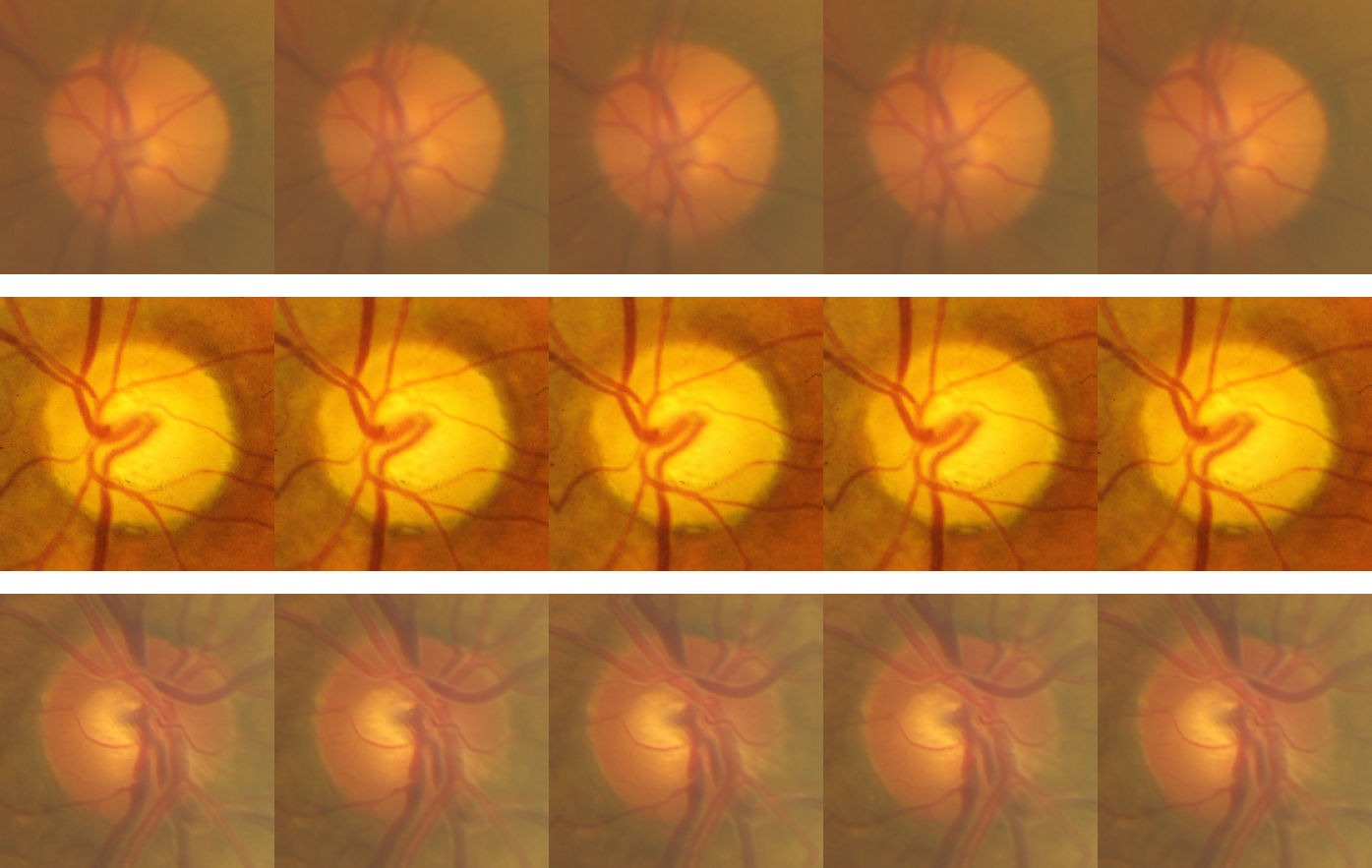


Supplemental Figure 7. Sample output of counter clockwise rotation augmentation. The first column is the unaltered image. Subsequent columns are random applications of the augmentation.


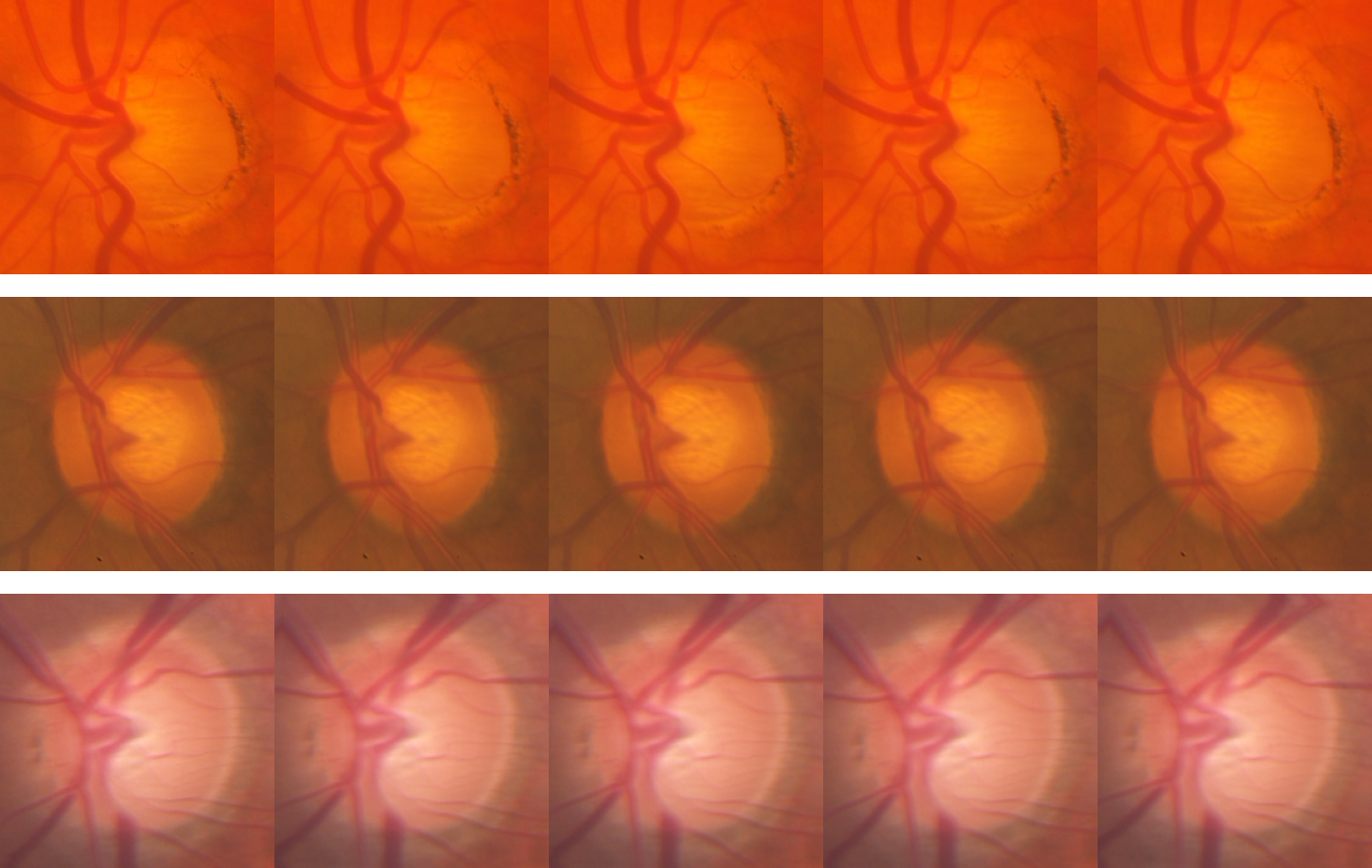


Supplemental Figure 8. Sample output of clockwise rotation augmentation. The first column is the unaltered image. Subsequent columns are random applications of the augmentation.


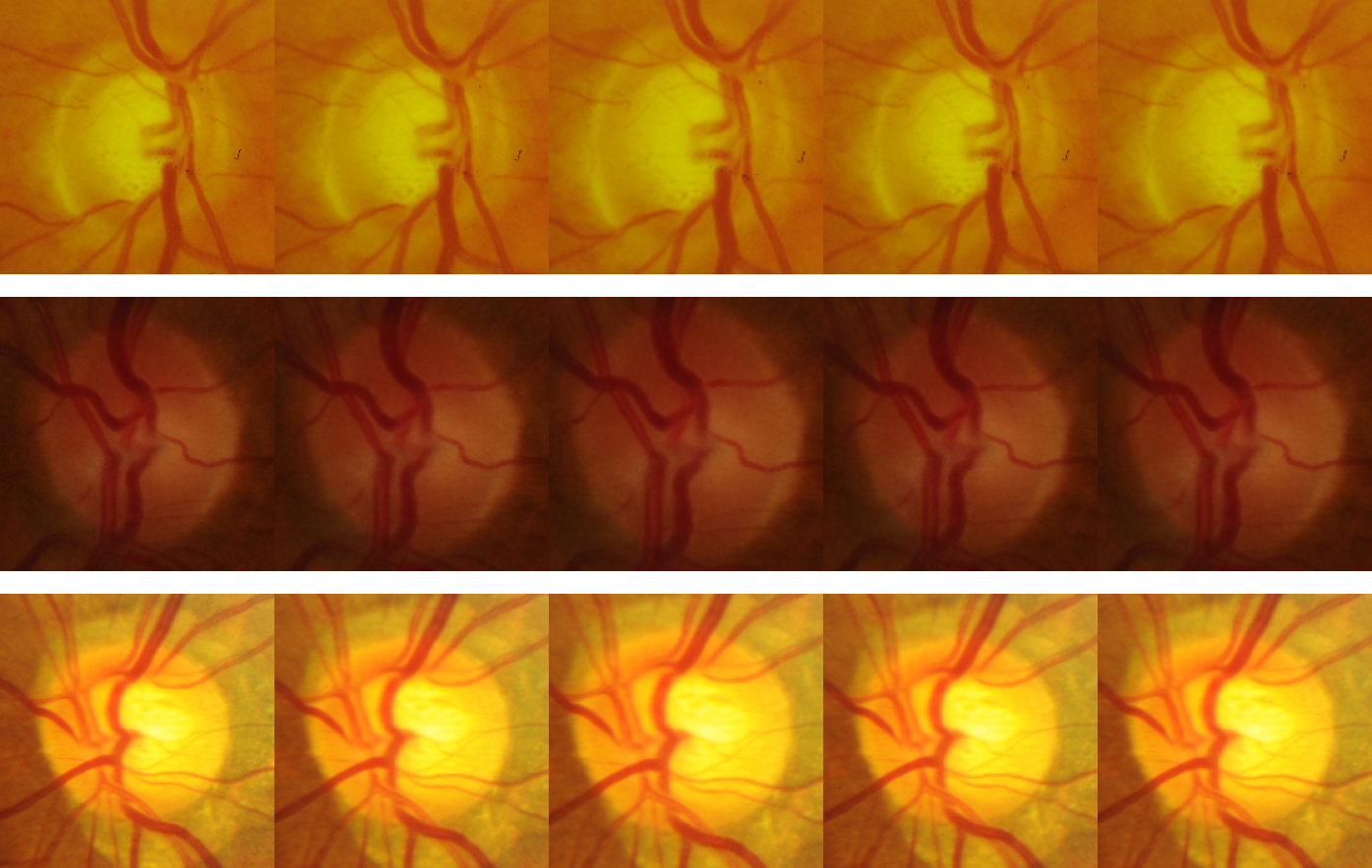


Supplemental Figure 9. Sample output of zoom augmentation. The first column is the unaltered image. Subsequent columns are random applications of the augmentation.
